# A Rare Case of Ectocervical Pregnancy and Its Successful Management: A Case Report and Review of the Literature

**DOI:** 10.7759/cureus.52388

**Published:** 2024-01-16

**Authors:** Alokananda Ray, Sarita Kumari, Pooja Lal

**Affiliations:** 1 Obstetrics and Gynaecology, Tata Main Hospital, Jamshedpur, IND

**Keywords:** conservative management, surgical excision, methotrexate, ectocervical pregnancy, ectopic pregnancy

## Abstract

Cervical pregnancy is a very rare form of ectopic pregnancy, which can be life-threatening due to the potential risk of massive haemorrhage. The most likely site of cervical implantation is within the endocervical canal. We report here an unusual and another possible site of cervical pregnancy on the surface of the ectocervix (portio). The patient presented with vaginal bleeding after a period of six weeks of amenorrhea and a positive urinary pregnancy test. Clinical examination was suggestive of a cervical mass on the surface of the portio, which was successfully managed by local excision and the application of haemostatic sutures. Histopathology of the mass was suggestive of trophoblasts amidst cervical epithelium and stroma, which was cytokeratin positive in immunohistochemical staining, confirming the diagnosis of cervical ectopic pregnancy on the portio. Postoperatively, the patient recovered well and beta-human chorionic gonadotropin (βhCG) normalised within two weeks. Thus, the surface of the ectocervix is another possible site of cervical pregnancy, which can be successfully managed by total excision of the ectopic mass and local haemostatic measures.

## Introduction

Ectopic pregnancy is defined as a clinical condition when the developing product of conception implants at a site other than the uterine endometrial cavity. The incidence of ectopic pregnancy has been reported as 0.25%-2%, of which most are located in the fallopian tube. Other rarer sites include the ovaries, intra-abdominally, previous caesarean section scar, and the cervix [1,2]. Cervical ectopic pregnancies are extremely rare, accounting for <1% of all ectopic pregnancies with nidation mostly occurring within the endocervical canal [3]. We report here an extremely unusual but another possible site of cervical pregnancy, i.e., on the surface of the portio or ectocervix (a location that has not been described very well in the literature), and its successful management.

## Case presentation

A 19-year-old nulliparous woman presented with a history of painless vaginal bleeding for one week, following a period of one and a half months of amenorrhea. The bleeding had become heavier, with mild lower abdominal pain over the past two to three days. Her previous cycles were regular and normal in duration and amount. She was not practising any contraceptive methods, and she had no other significant past or personal history.

On examination, her vitals were stable and vaginal bleeding was mild. Per speculum examination revealed a fleshy reddish brown polypoidal mass, bleeding to touch at 10 to 11 o’clock position on the ectocervix 6 mm away from the external cervical os. A urinary pregnancy test was positive, with a corresponding serum beta-human chorionic gonadotropin (βhCG) level of 432 mIU/ml. Transabdominal ultrasound of the pelvis revealed a bulky uterus measuring 9 x 6 x 4 cm with closed internal os, the cervical length was 3 cm, endometrial thickness (ET) was 1.2 cm, and bilateral ovaries and tubes were normal. Both the uterine cavity and cervical canal were empty, with no visible gestational sac. There was no collection in the pouch of Douglas (POD). Transvaginal ultrasonography was not done because the cervical mass was bleeding to the slightest of manipulation and touch.

**Figure 1:**
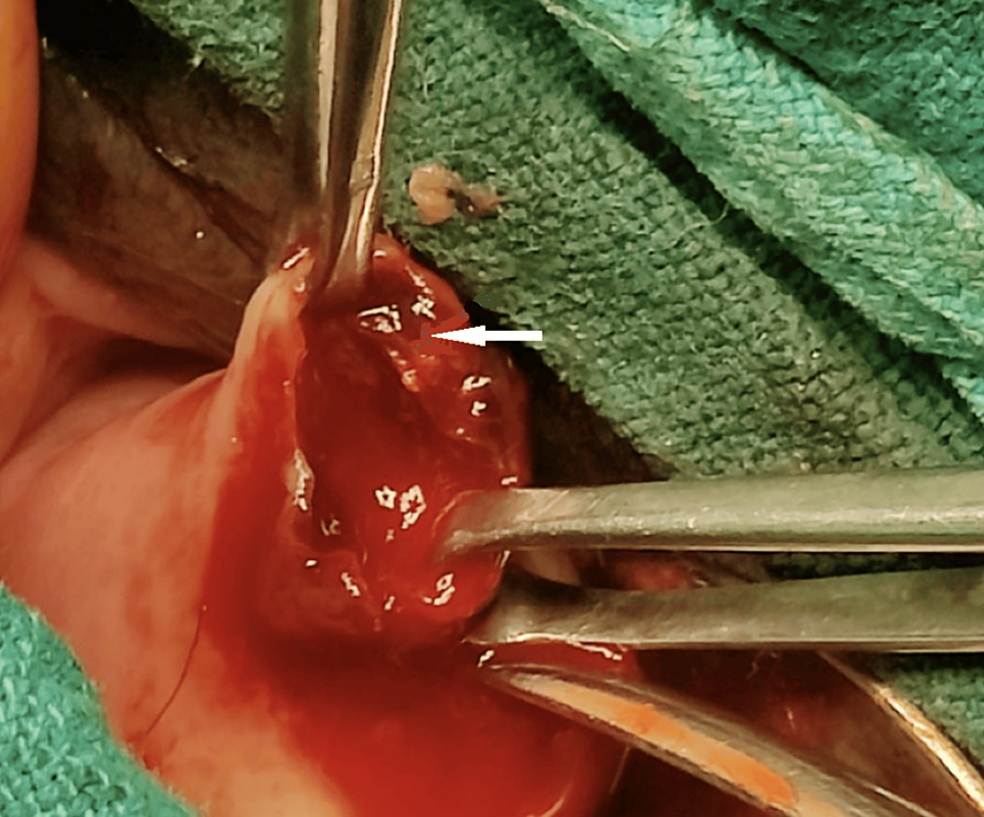
Fleshy mass at 10 to 11 o'clock position on the ectocervix.

Differential diagnoses of incomplete intrauterine miscarriage or very early intrauterine pregnancy with a cervical polyp or an ectopic cervical pregnancy on the portio were considered at this point in time. After informed consent, the patient was taken up for examination under anaesthesia and removal of the cervical mass with exploration of the uterine cavity and cervix, as she was not desirous of a pregnancy. On examination under anaesthesia, the cervix appeared long and tubular. A reddish brown soft mass of 2 x 3 cm was seen, 6 mm away from the external os attached to the ectocervix at the 10 to 11 o’clock position (marked with an arrow in Figure 1).

**Figure 2:**
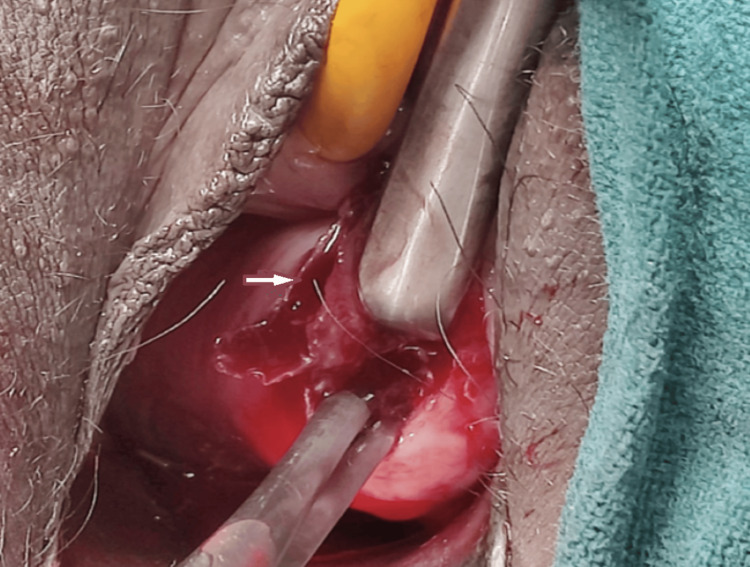
Ectocervical mass excised completely.

**Figure 3:**
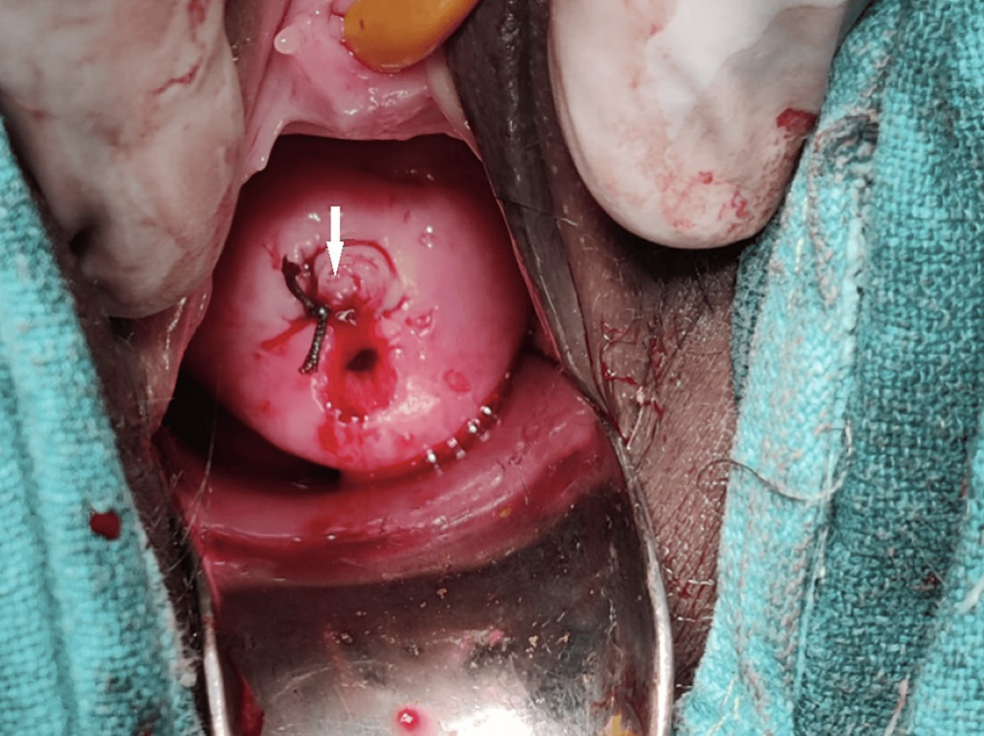
Haemostatic sutures applied to control bleeding from the excision site.

The bi-manual vaginal examination was suggestive of an anteverted bulky uterus, long tubular cervix with open external os, and normal bilateral fornices. The fleshy cervical mass was firmly adherent to the surface of the ectocervix, and digital manipulation of the mass led to an increase in vaginal bleeding. A small incision was given at the junction of the mass with the ectocervix, and tissue looking like products of conception was removed completely. Haemostatic sutures were applied to secure mild to moderate bleeding from the surgical site (marked with an arrow in Figures 2, 3).

The cervical os was dilated with serial mechanical dilatation followed by endocervical and endometrial curettage. All samples were sent for histopathological examination. The immediate postoperative period was uneventful, and the patient was discharged the following day in a stable condition with advice for weekly serial βhCG estimation.

**Figure 4:**
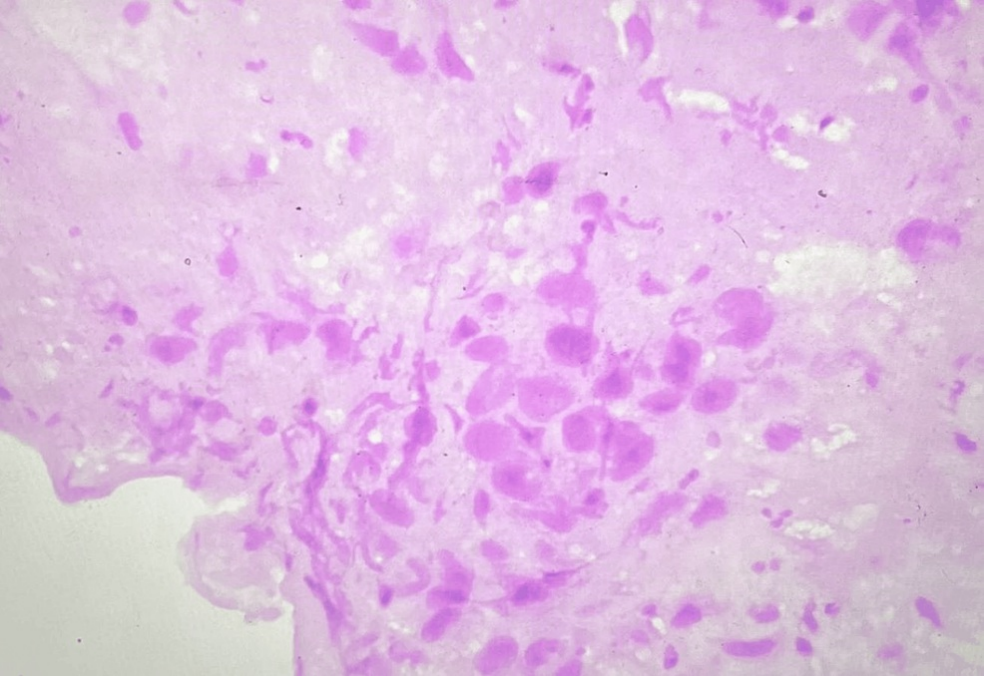
Histopathology of the cervical mass showing intermediate trophoblasts in a background of cervical epithelium and stroma (hematoxylin and eosin stain, original magnification x40).

Histopathology of the ectocervical mass was suggestive of trophoblastic tissue amidst cervical epithelium and stroma. The endometrial and endocervical biopsies were devoid of trophoblasts, confirming the diagnosis of cervical ectopic pregnancy on the surface of the portio or ectocervix (Figure 4). The intermediate trophoblast cells invading the cervical epithelium and stroma were further identified and confirmed by cytokeratin (CK) immunohistochemical staining, which suggested a cytokeratin immunoreactive score 4+ in lesional cells strongly consistent with trophoblastic tissue. The βhCG level was normalised to 3.5 mIU/ml (normal value < 5 mIU/ml) by the end of the second week. Both the immediate and late postoperative periods were uneventful, with satisfactory patient recovery.

## Discussion

Cervical pregnancy is a potentially life-threatening and rare site of extra-uterine pregnancy, occurring in 1:1000 to 1:18,000 deliveries, where the developing embryo usually implants in the endocervical canal or the fibro-muscular layer of the cervix [4,5]. We have described here an ectopic implantation on the surface of the exocervix, as another possible site of cervical pregnancy.

The cause of cervical pregnancy could be a combination of several factors like previous dilatation and curettage, Asherman’s syndrome, anatomical or structural abnormality of the uterus, immature uterine endometrial lining or endometrial inflammation not conducive to intrauterine implantation, previous caesarean section, previous cervical or uterine surgery, presence of intrauterine device (IUD) hastening the transfer of embryo from the uterine cavity to the cervix, cervical endometriosis, and assisted reproductive technology (ART) with embryo transfer [6,7]. The aetiology of the even rarer entity of the embryo implanting on the surface of the ectocervix as presented here in our case is uncertain, and fertilisation within the cervical canal near the external os or on the surface of the cervix with subsequent implantation on the surface of the portio could be a possible cause [8].

The clinical presentation of cervical pregnancy is usually painless uterine bleeding after a variable period of amenorrhea, as was reported in our case. However, lower abdominal pain and urinary symptoms may be seen in advanced cervical pregnancies presenting in the late 1st or early 2nd trimester of pregnancy [6]. The clinical criteria for diagnosing cervical ectopic pregnancy include the following: (1) a period of amenorrhea followed by painless vaginal bleeding; (2) a soft and enlarged cervix, which may be larger than the size of the uterine corpus; (3) products of conception entirely within the endocervical canal and firmly attached to the wall of the cervix; and (4) closed internal os and partially open external os [9]. Ultrasonography, preferably transvaginal (TVS), typically shows an empty uterine cavity and a gestational sac with or without a fetus, within the cervical canal, invading the anterior or the posterior wall of the cervix. The dilated cervix and empty endometrial cavity give an appearance of an hourglass. Peri-trophoblastic vascularity in the endocervix and the absence of sliding signs are suggestive of implantation within the cervical canal [10]. None of these ultrasound findings, however, could be elicited in our patient, as the developing pregnancy was on the surface of the ectocervix, which was clinically evident as a fleshy reddish brown polypoidal mass, at 10 to 11 o’clock position on the ectocervix 6 mm away from the external cervical os.

There are no specific guidelines for the management of cervical pregnancy. Due to its rarity, treatment recommendations are mainly based on case reports or small case series. These treatment options mainly depend upon the haemodynamic condition of the patient, period of gestation at diagnosis, baseline βhCG level, crown-rump length (CRL) of the fetus, presence or absence of fetal cardiac activity, response to initial treatment, and patient’s desire to preserve fertility.

Treatment options for cervical ectopic pregnancy include medical management or surgical removal of the ectopic gestation. Similar to tubal ectopic pregnancy, early diagnosis of cervical pregnancy, lower βhCG levels, and absence of fetal cardiac activity are likely to have a successful outcome with a weight-based dosage of injection methotrexate (MTX) [11]. In the presence of fetal cardiac activity, and or gestational age greater than nine weeks, intra-amniotic injection of potassium chloride (KCl) for feticide followed by systemic or local injection of MTX can be attempted [12]. Other than KCl and MTX, local injections of cyclophosphamide, etoposide, actinomycin D, and prostaglandin F2α have also been reported in the literature [8]. The advantage of medical management is that they are fertility-sparing and thus preferred by patients who are mostly young and desirous of future pregnancies.

Surgical management of cervical pregnancy, with the intent to preserve the uterus and future fertility, involves dilatation and evacuation (D&E) of the cervical products of conception. The major complications associated with this method of treatment include cervical trauma and/or torrential haemorrhage. Trauma to the cervix can be prevented by the use of misoprostol for cervical priming prior to mechanical cervical dilatation. The cervix, which consists mostly of fibrous and <20% smooth muscle tissue, responds weakly to uterotonic agents and mechanical tamponade. Bleeding encountered in cervical evacuation can be controlled by the use of local injection of vasopressin and systemic injection of tranexamic acid [13,14], or by haemostatic procedures like cervical cerclage, surgical ligation of cervical, uterine, or internal iliac arteries, or by uterine artery embolisation (UAE), which can be performed primarily or in conjunction with endovascular arterial occlusion balloon placement [15,16]. Regardless of the method, all women undergoing conservative medical or surgical management should be followed up till complete resolution of the ectopic pregnancy, defined as βhCG levels < 5 mIU/ml. Hysterectomy is performed as the last resort in case of intractable bleeding or if the above-mentioned treatment protocols fail or are unavailable.

In our case, the ectopic gestation was on the surface of the ectocervix, which was surgically excised followed by the application of haemostatic sutures to control the bleeding. The ectopic tissue could be removed completely as suggested by a normal βhCG level by the end of two weeks following the procedure. Systemic or local chemotherapeutic agents, suction curettage, or uterine devascularisation methods, which are fertility-conserving treatment options for endocervical ectopic pregnancy, were not required in our case. Postoperatively, the patient recovered well. Similar favourable outcomes with surgical excision of cervical pregnancy on the surface of the ectocervix have been reported in the literature [7,8]. However, because of its extreme rarity, more often than not, it is mistaken for other common cervical lesions like endometriosis or bleeding polyp, and the final diagnosis of ectopic pregnancy on the surface of the portio is confirmed, after surgical removal and histopathological assessment of the cervical mass [7,8].

## Conclusions

Cervical ectopic pregnancies are extremely rare, accounting for <1% of all ectopic pregnancies with implantation occurring mostly within the endocervical canal. We have reported here another possible site of cervical pregnancy, namely, the surface of the portio or ectocervix, which can be successfully treated with complete surgical excision of the ectopic tissue and local haemostatic measures.
